# Correction to Figures: A Reply to Hwang and Peli (2014)

**DOI:** 10.1177/2041669517723929

**Published:** 2017-08-10

**Authors:** Zhongpai Gao

**Affiliations:** Institute of Image Communication and Information Processing, Shanghai Jiao Tong University, Shanghai, China; Schepens Eye Research Institute, Massachusetts Eye and Ear Infirmary, Department of Ophthalmology, Harvard Medical School, Boston, MA, USA

**Keywords:** 3D perception, motion sickness, stereoscopic display, 3D display

## Abstract

In Hwang and Peli (2014), few errors occurred in computing the angular disparities. The direction of peripheral depth distortion (the angular disparity differences between what it is in real-world 3D viewing and S3D viewing) is reversed when the computational errors were corrected, making the perception of the peripheral depth to be expanded, not compressed. This reply points to the error and provides the corrected figures. Correcting these errors does not affect the general conclusion that S3D viewed on single screen display induces peripheral depth distortion which may be a cause of visually induced motion sickness.

There were computational errors in generating Figures 9 and 10 of the Hwang and Peli (2014) paper. The corrected figures are presented later as Figures 1 and 2, respectively. The computation of the angular disparity (AD) and the explanation of the computational error are presented in Appendix.

**Figure 1.**
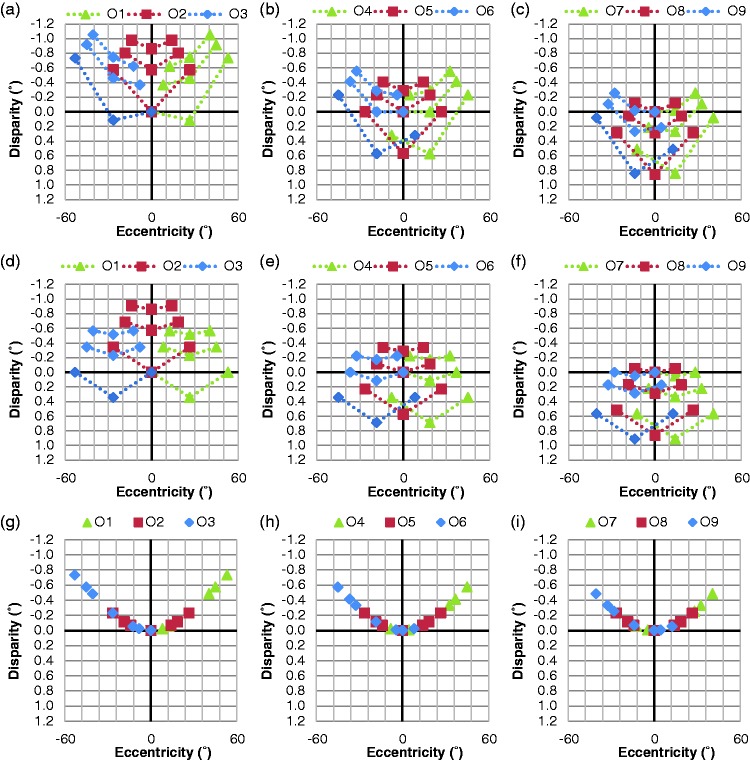
This figure should replace the Figure 9 of the Hwang and Peli (2014) paper. The effect of gaze shifts on AD as a function of eccentricity while the viewer’s head remains centered. The ADs of all nine objects are shown for each gaze (fixation) position. In the first two rows (panels a to f), legend symbols distinguish gaze position, not objects rows, with all nine objects per gaze having the same symbol. Panels (a), (b), and (c) show the ADs with S3D viewing, each with three gaze positions overlaid (for fixations on the objects in first row, second row, and third row, respectively). Panels (d) to (f) show the corresponding ADs during natural viewing. Panels (g) to (i) plot the arithmetic difference between the S3D and natural ADs as a function of VE, with symbols representing gazed objects, O1 to O9. The amount of the depth distortion is largely independent of aiming distance (vergence angle), but is substantial at larger VEs.

**Figure 2.**
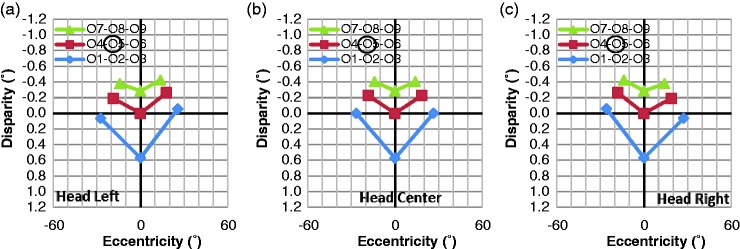
This figure should replace the first row of the Figure 10 of the Hwang and Peli (2014) paper. Distribution of ADs and virtual locations of objects viewing in S3D when the eyes’ position has shifted (a) −0.2 m, (b) 0.0 m, and (c) 0.2 m from the center while fixating on the center object (O5).

These errors resulted in incorrect depiction of the perceptual depth distortion from viewer’s perspective, where the disparity differences between the real-world 3D viewing and S3D viewing should be increased in negative (uncrossed) direction (as shown in Figure 1(g) to (i)), instead of positive (crossed) direction (as shown in Figure 9 (g) to (i) of the paper), as the eccentricity is increased.

This corrected depiction indicates a depth expansion (not compression) in the viewer’s peripheral field, which makes any motion in depth in the peripheral area to be perceived slower than it should be.

The same errors also affected a few panels in Figure 10 of the Hwang and Peli (2014) paper, which illustrated the effect of viewer’s lateral head position shift in S3D viewing. Although the magnitude of distortion was changed in the corrected disparity structure depicted (Figure 2), the main point of the paper, the perception of world rotation following the viewer’s head position shift, is still well supported. Note that the viewer’s impression of the world rotation mentioned in the Hwang and Peli (2014) paper, in fact, is a sheering of the 3D scene in depth direction.

Although the direction of peripheral depth distortion has been reversed and the amount of the distortion caused by viewer’s lateral head shift have been corrected, these changes are not affecting the general conclusion of the paper, that the peripheral depth distortion and viewer’s position shift while viewing S3D may induce non-rigidity of the depicted world, and it may be a likely source of the visually induced motion sickness.

## Appendix

### Angular Disparity Calculation

The angular disparities (ADs) in Figure 1(a) to (c) were obtained by assuming that the two capturing cameras were converged to each of the nine objects and the viewer of the captured S3D scene was looking at the center of the screen. The ADs in Figure 1(d) to (f) were obtained by assuming that the viewer was directly fixating at each of the nine objects in natural viewing condition.

In natural viewing condition, the ADs of object locations in 3D space while fixating on a point are defined in section 3.3 of the Hwang and Peli (2014) as:

(A1) $${\mathrm{VE}}_{L,i}=\text{atan}(\frac{O_{i,x}-E_{L,x}}{O_{i,y}-E_{L,y}})-\text{atan}(\frac{O_{F,x}-E_{L,x}}{O_{F,y}-E_{L,y}})$$

(A2) $${\mathrm{VE}}_{R,i}=\text{atan}(\frac{O_{i,x}-E_{R,x}}{O_{i,y}-E_{R,y}})-\text{atan}(\frac{O_{F,x}-E_{R,x}}{O_{F,y}-E_{R,y}})$$

(A3) $${\mathrm{AD}}_{i}={\mathrm{VE}}_{L,i}-{\mathrm{VE}}_{R,i}$$

where *VE_L,i_*, *VE_R,i_*: visual eccentricity of object *i*, as seen in the left and right eye, respectively; *AD_i_*: angular disparity of object *i*, *O_F,x_*,* O_F,y_*,* O_i,x_*,* O_i,y_*: *x* and *y* coordinates of the fixated object *F* and object *i*; and *E_L,x_*,* E_L,y_*,* E_R,x_*,* E_R,y_*: *x* and *y* coordinates of left and right eye nodal points.

In S3D viewing condition, the object that the cameras converged to is (*O_F,x_*, *O_F,y_*). After the scene was captured, the left and right images were presented on the S3D display at a distance *d*. The object that the cameras converged to is always shown at (0, *d*). For the other objects (*O_i,x_*, *O_i,y_*), the on-screen coordinates for the left and right views are shown at (*S_L,i,x_*, *d*) and (*S_R,i,x_*, *d*). The horizontal components can be expressed by

(A4) $$S_{L,i,x}=\text{d}\cdot \text{tan}({\mathrm{VE}}_{L,i})$$

(A5) $$S_{R,i,x}=\text{d}\cdot \text{tan}({\mathrm{VE}}_{R,i})$$

Then, the VEs and AD of the objects in S3D can be formulated as following

(A6) $${\mathrm{VES}}_{L,i}=\text{atan}(\frac{S_{L,i,x}-E_{L,x}}{d-E_{L,y}})-\text{atan}(\frac{0-E_{L,x}}{d-E_{L,y}})$$

(A7) $${\mathrm{VES}}_{R,i}=\text{atan}(\frac{S_{R,i,x}-E_{R,x}}{d-E_{R,y}})-\text{atan}(\frac{0-E_{R,x}}{d-E_{R,y}})$$

(A8) $${\mathrm{ADS}}_{i}={\mathrm{VES}}_{L,i}-{\mathrm{VES}}_{R,i}$$

where *VES_L,i_*, *VES_R,i_*: visual eccentricity of object *i* in S3D, respectively, and* ADS_i_*: angular disparity of object *i* in S3D.

In Hwang and Peli (2014) paper, I noted that the retinal visual eccentricities (*VES_L,i_* and *VES_R,i_*) of the objects projected on the single screen display were miscalculated when two of the figures were generated. Specifically, it seems that the *x* and *y* coordinates of left and right eye nodal points (*E_L,x_*, *E_L,y_*, *E_R,x_*, and *E_R,y_*) summed (rather than subtracted from) the object coordinates in Equations (A6) and (A7).
